# Detailed data from experimentally-induced mastitis in ewes, with the aim to evaluate cathelicidin-1 in milk

**DOI:** 10.1016/j.dib.2020.105259

**Published:** 2020-02-08

**Authors:** Angeliki I. Katsafadou, George Th. Tsangaris, Natalia G.C. Vasileiou, Katerina S. Ioannidi, Athanasios K. Anagnostopoulos, Charalambos Billinis, Ilektra A. Fragkou, Elias Papadopoulos, Vasia S. Mavrogianni, Charalambia K. Michael, M. Filippa Addis, George C. Fthenakis

**Affiliations:** a Veterinary Faculty, University of Thessaly, 43100 Karditsa, Greece; b Proteomics Research Unit, Biomedical Research Foundation of Academy of Athens, 11527 Athens, Greece; c Laboratory of Parasitology and Parasitic Diseases, Faculty of Veterinary Medicine, Aristotle University of Thessaloniki, 54124 Thessaloniki, Greece; d Dipartimento di Medicina Veterinaria, Università degli Studi di Milano, Via G. Celoria 10, 20133 Milan, Italy

**Keywords:** Biomarker, Diagnosis, Mastitis, Sheep, Somatic cell counts

## Abstract

Bacteriological, cytological and proteomics data have been obtained from ewes in two experiments, after intramammary challenge with *Mannheimia haemolytica* or *Staphylococcus chromogenes*. Animals were sampled before and sequentially after challenge. Conventional techniques were employed for bacterial isolation and somatic cell counting in milk samples; milk whey samples were subjected to proteomics evaluation by using two-dimensional gel electrophoresis and MALDI-TOF mass spectrometry. There was a correlation between leucocyte content and cathelicidin-1 spot densities in milk samples, although the protein was detected in milk earlier than the increase in leucocyte content. There was also a significant association between presence of mastitis in a mammary gland and detection of cathelicidin-1 in the respective milk sample; the degree of association was greater during the first 24 h post-inoculation. The data are further discussed in the research article “Detection of cathelicidin-1 in the milk as an early indicator of mastitis in ewes” [1].

**Table:** Specifications Table

Subject area	*Veterinary Science and Veterinary Medicine*
More specific subject area	*Sheep, Infectious Diseases*
Type of data	*Tables, figures*
How data was acquired	*Experimental infection of ewes* *Bacteriological and cytological examination of milk samples* *Proteomics analysis of milk whey samples by 2-DE and MALDI-TOF MS*
Data format	*Raw and analysed*
Experimental factors	*Milk samples from ewes with experimentally-induced mastitis (Mannheimia haemolytica or Staphylococcus chromogenes) were collected and analysed, in order to evaluate the possibility of diagnosing mastitis by detection of cathelicidin-1 therein*
Experimental features	*Experimental induction of mastitis to confirm disease status of ewes* *Collection of first sample 3 h post-challenge to guarantee close monitoring of the course of disease*
Data source location	*Karditsa, Greece, Veterinary Faculty, University of Thessaly*
Data accessibility	*All data are with article*
Related research article	*A.I. Katsafadou, G.T. Tsangaris, N.G.C. Vasileiou, K.S. Ioannidi, A.K. Anagnostopoulos, C. Billinis, I.A. Fragkou, E. Papadopoulos, V.S. Mavrogianni, C.K. Michael, M.F. Addis, G.C. Fthenakis, Detection of cathelicidin-1 in the milk as an early indicator of mastitis in ewes.Pathogens 8 (2019) 270*

**Table:** 

**Value of the Data** • This is the only dataset available from experimentally induced mastitis, detailing cathelicidin-1 presence in milk, with early start of monitoring post-challenge, to fully evaluate the course of cathelicidin-1 presence in milk. • The data can be used by researchers working in the development of diagnostic techniques for mastitis, based on detection of cathelicidin-1 in milk.

## 1 Data

**Fig. 1:**
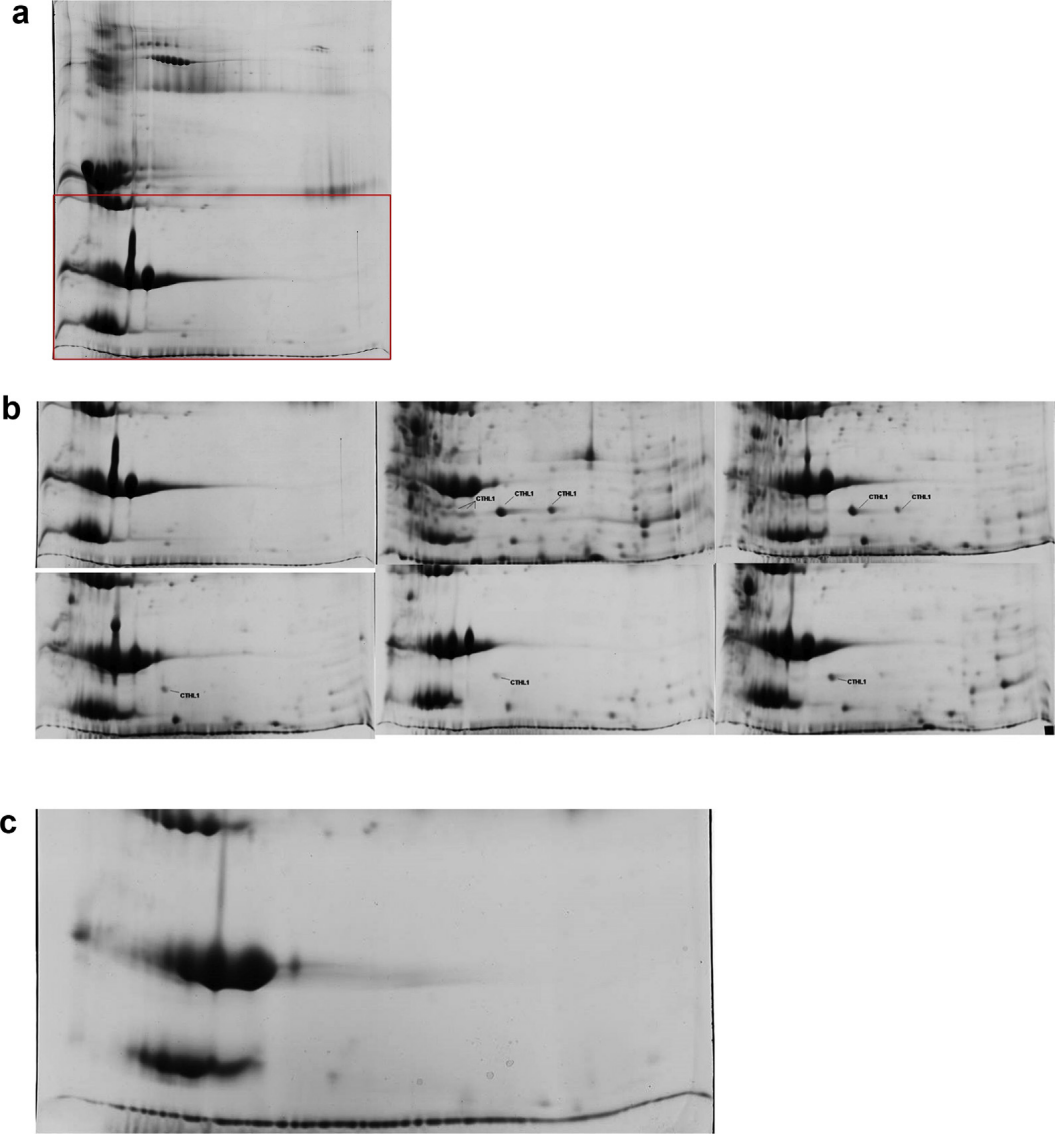
2-DE gels with annotation of cathelicidin-1, obtained from milk samples (whey) collected from the inoculated side of the udder of a ewe before or after inoculation of the ipsilateral teat with *M. haemolytica* (a, b) or from the uninoculated side of the udder of the same ewe (c) (protein identification by MALDI-TOF MS) (experiment 1). (a) 2-DE gel obtained from a whey sample before challenge, from the inoculated side of the udder of a ewe; the area in red indicates the region of the gels shown in detail in (b) and (c). (b) Region of 2-DE gels obtained from whey samples before or sequentially after challenge, from the inoculated side of the udder of a ewe; from top left to the right and from bottom right to the right: before inoculation (D0), 12 h after inoculation (D0+12 h), 1 d after inoculation (D1), D2, D3, D4. (c) Region of 2-DE gel obtained from whey sample 12 h after inoculation of the contralateral side of the udder. Horizontal axis: isoelectric point 3 to 10 (non-linear) from left to right; vertical axis: molecular weight 10–100 kDa (non-linear) from bottom to top.

**Fig. 2:**
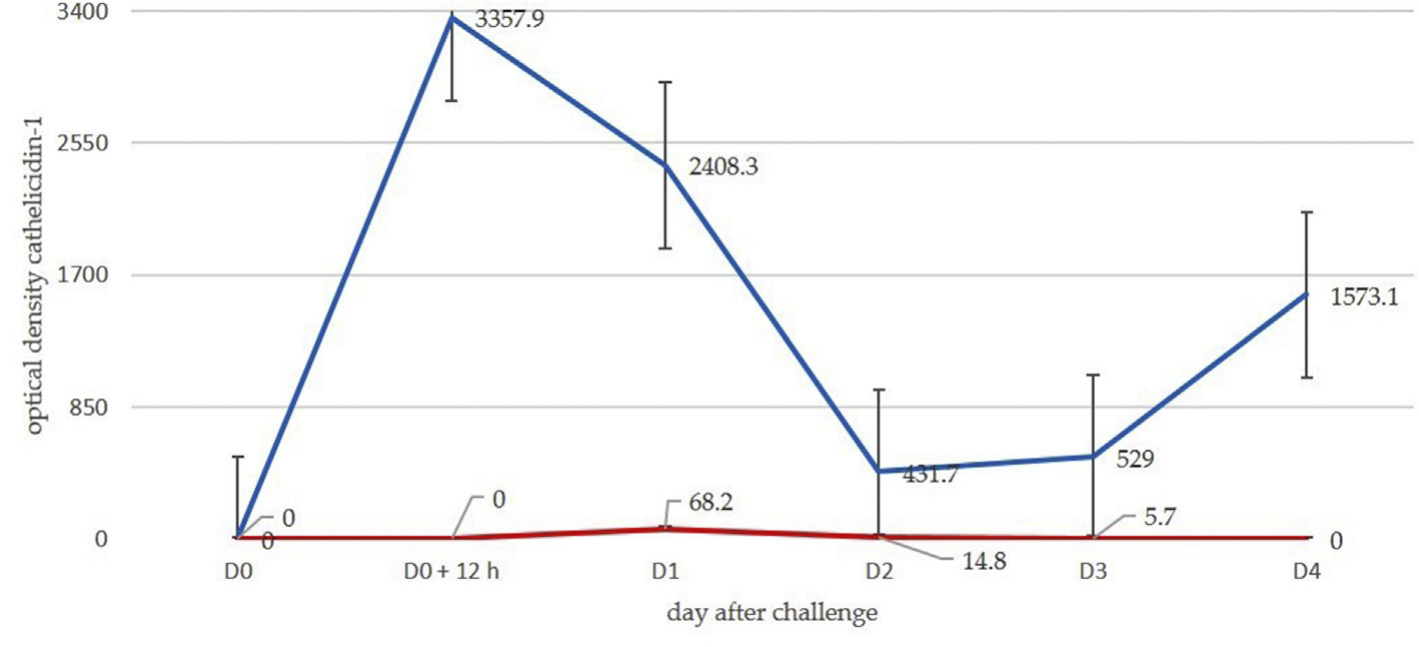
Mean spot densities of cathelicidin-1 in 2-DE gels obtained from sequential milk samples from inoculated (red line) or uninoculated (blue line) side of the udder, subsequently to inoculation of one teat with *M. haemolytica* (protein identification by MALDI-TOF MS) (experiment 1). Experiment 1: deposition of *M. haemolytica* into the teat duct.

**Fig. 3:**
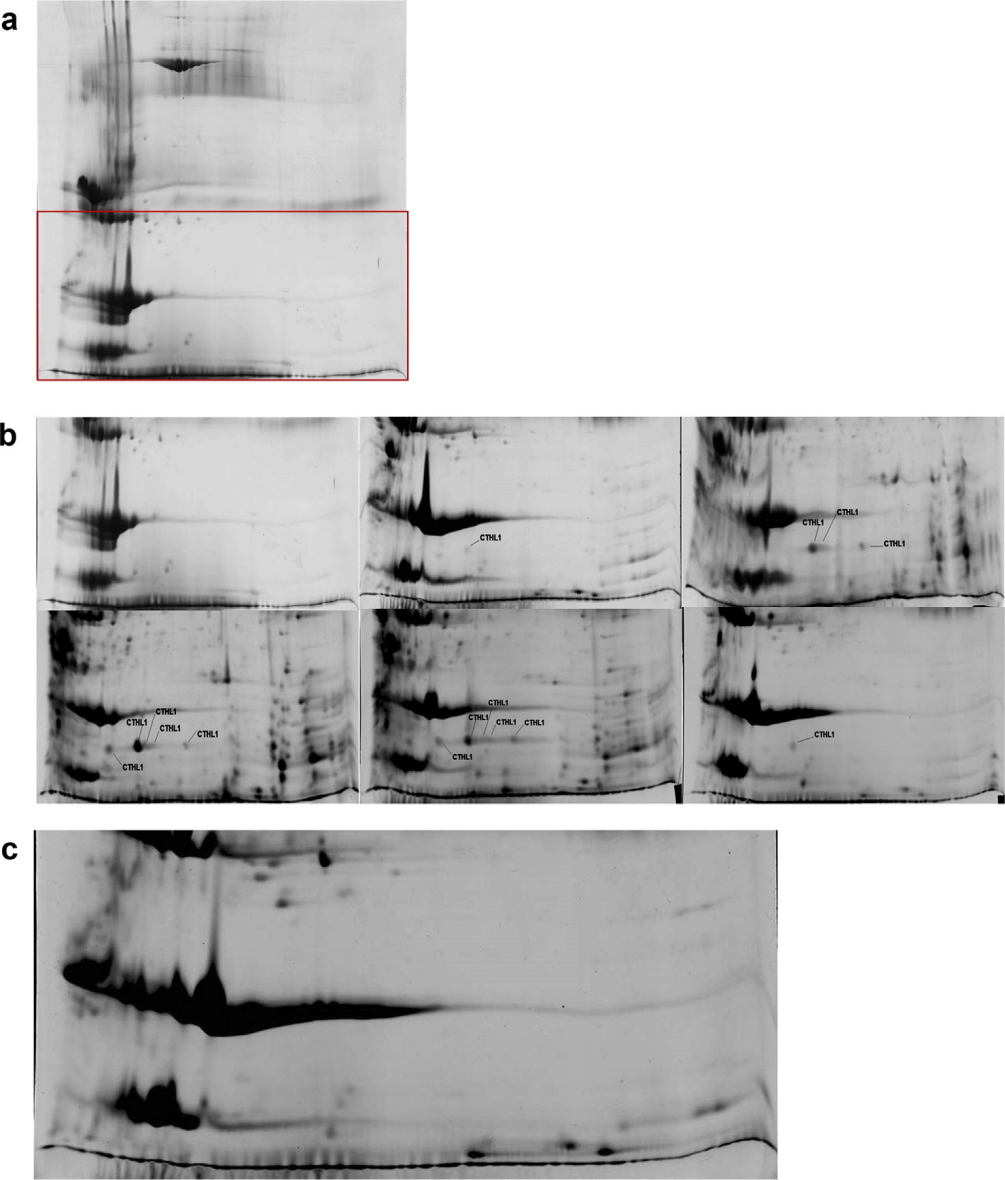
2-DE gels with annotation of cathelicidin-1, obtained from milk samples (whey) collected from the inoculated side of the udder of a ewe before or after inoculation of the ipsilateral gland with *S. chromogenes* (a, b) or from the uninoculated side of the udder (c) (protein identification by MALDI-TOF MS) (experiment 2). (a) 2-DE gel obtained from whey sample before challenge, from the inoculated side of the udder of a ewe; the area in red indicates the region of the gels shown in detail in (b) and (c). (b) Region of 2-DE gels obtained from whey samples before or sequentially after challenge, from the inoculated gland; from top left to the right and from bottom right to the right: before inoculation (D0), 3 h after inoculation, 6 h after inoculation, 9 h after inoculation, 12 h after inoculation, 24 h after inoculation. (c) Region of 2-DE gel obtained from pooled whey samples, from the contralateral to inoculated gland. Horizontal axis: isoelectric point 3 to 10 (non-linear) from left to right; vertical axis: molecular weight 10–100 kDa (non-linear) from bottom to top.

**Fig. 4:**
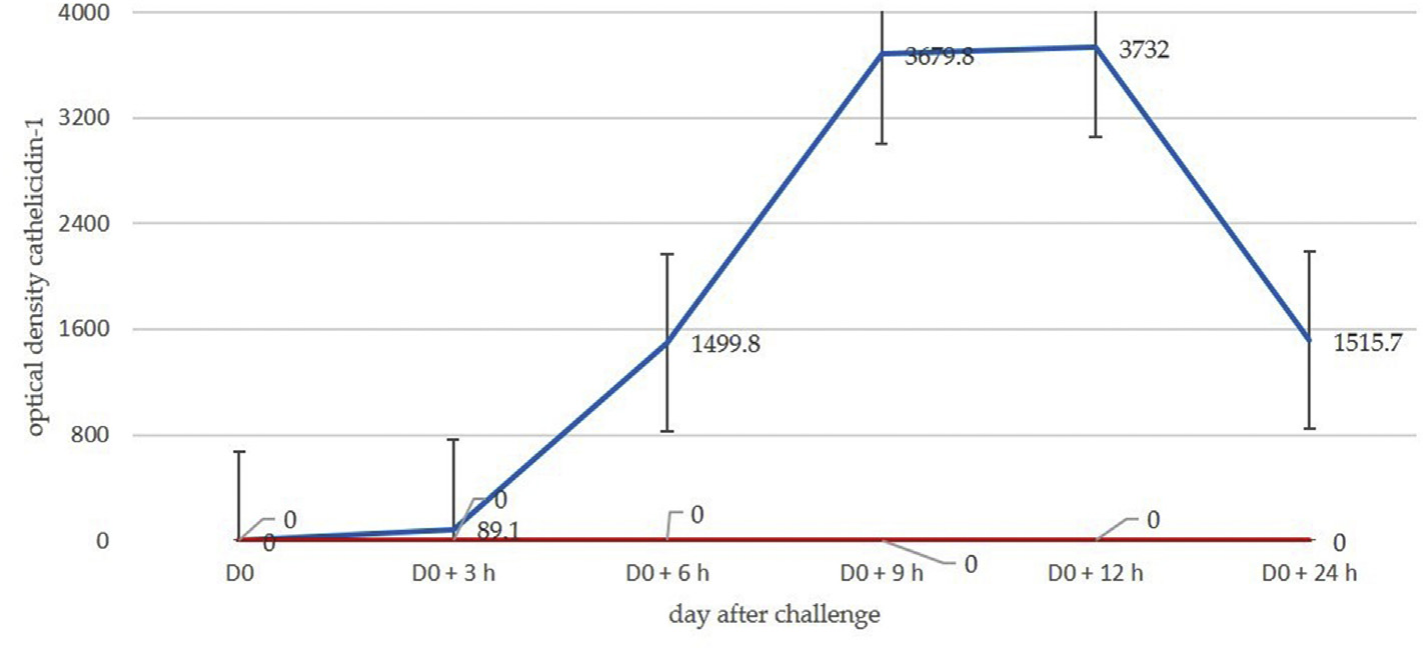
Mean spot densities of cathelicidin-1 in 2-DE gels obtained from sequential milk samples from inoculated (straight line) or uninoculated (dotted line) side of the udder, subsequently to inoculation of one gland with *M. haemolytica* or *S. chromogenes* (experiment 2). Experiment 2: inoculation of *M. haemolytica* or *S. chromogenes* into the mammary gland cistern.

In two experiments, we performed intramammary challenge of ewes with *Mannheimia haemolytica* or *Staphylococcus chromogenes*; subsequently, mastitis was induced [1], as confirmed by clinical, microbiological and cytological findings (Table 1, Table 2, Table 3, Table 4). Presence of cathelicidin-1 in milk was also evaluated (Table 5, Table 6, Fig. 1, Fig. 2, Fig. 3, Fig. 4).

**Table 1:** Detailed data of isolation of *M. haemolytica* or *S. chromogenes* from milk samples of ewes after intramammary inoculation (performed on D0, after the respective sampling).

Ewe no.	D0		D0 + 12 h		D1		D2		D3		D4	
	i. s.	c. s.	i. s.	c. s.	i. s.	c. s.	i. s.	c. s.	i. s.	c. s.	i. s.	c. s.
(a) Experiment 1 (deposition of *M. haemolytica* into the teat duct)												
1	–	–	–	–	+	–	+	–	+	–	+	–
2	–	–	–	–	+	–	–	–	–	–	NA	NA
3	–	–	+	–	+	–	+	–	+	–	–	–
4	–	–	–	–	+	–	+	–	–	–	NA	NA
5	–	–	–	–	+	–	–	–	–	–	NA	NA

Ewe no.	D0		D0 + 3 h		D0 + 6 h		D0 + 9 h		D0 + 12 h		D1	
	i. s.	c. s.	i. s.	c. s.	i. s.	c. s.	i. s.	c. s.	i. s.	c. s.	i. s.	c. s.
(b) Experiment 2 (inoculation of *M. haemolytica* [ewes 11–13] or *S. chromogenes* [ewes 14–16] into the mammary gland cistern)												
11	–	–	+	–	+	–	+	–	+	–	+	–
12	–	–	+	–	+	–	+	–	+	–	+	–
13	–	–	+	–	+	–	+	–	+	–	+	–
14	–	–	+	–	+	–	+	–	+	–	+	–
15	–	–	+	–	+	–	+	–	+	–	+	–
16	–	–	+	–	+	–	+	–	+	–	+	–

i. s.: inoculated side of the udder, c. s.: uninoculated side of the udder.

+: isolation of the challenge pathogen, -: no bacterial isolation.

**Table 2:** Detailed data of California Mastitis Test scores in milk samples of ewes after intramammary inoculation (performed on D0, after the respective sampling).

Ewe no.	D0		D0 + 12 h		D1		D2		D3		D4	
	i. s.	c. s.	i. s.	c. s.	i. s.	c. s.	i. s.	c. s.	i. s.	c. s.	i. s.	c. s.
(a) Experiment 1 (deposition of *M. haemolytica* into the teat duct)												
1	neg.	neg.	2	neg.	3	neg.	3	neg.	2	neg.	2	neg.
2	neg.	neg.	2	neg.	2	neg.	3	neg.	2	neg.	NA	NA
3	neg.	neg.	1	neg.	3	neg.	3	neg.	2	neg.	trace	neg.
4	neg.	neg.	2	neg.	2	neg.	2	neg.	2	neg.	NA	NA
5	neg.	neg.	2	neg.	2	neg.	1	neg.	trace	neg.	NA	NA

Ewe no.	D0		D0 + 3 h		D0 + 6 h		D0 + 9 h		D0 + 12 h		D1	
	i. s.	c. s.	i. s.	c. s.	i. s.	c. s.	i. s.	c. s.	i. s.	c. s.	i. s.	c. s.
(b) Experiment 2 (inoculation of *M. haemolytica* [ewes 11–13] or *S. chromogenes* [ewes 14–16] into the mammary gland cistern)												
11	neg.	neg.	neg.	neg.	1	neg.	1	neg.	2	neg.	2	neg.
12	neg.	neg.	neg.	neg.	1	neg.	1	neg.	2	neg.	2	neg.
13	neg.	neg.	neg.	neg.	neg.	neg.	2	neg.	2	neg.	2	neg.
14	neg.	neg.	trace	neg.	2	neg.	2	neg.	2	neg.	2	neg.
15	neg.	neg.	neg.	neg.	trace	neg.	1	neg.	2	neg.	2	neg.
16	neg.	neg.	trace	neg.	2	neg.	2	neg.	2	neg.	2	neg.

i. s.: inoculated side of the udder, c. s.: uninoculated side of the udder.

CMT scores: negative (neg.), trace, 1, 2, 3.

**Table 3:** Detailed data of somatic cell counts in milk samples of ewes after intramammary inoculation (performed on D0, after the respective sampling). Experiment 2 (inoculation of *M. haemolytica* [ewes 11–13] or *S. chromogenes* [ewes 14–16] into the mammary gland cistern).

Ewe no.	D0		D0 + 3 h		D0 + 6 h		D0 + 9 h		D0 + 12 h		D1	
	i. s.	c. s.	i. s.	c. s.	i. s.	c. s.	i. s.	c. s.	i. s.	c. s.	i. s.	c. s.
11	429	397	419	398	796	398	769	397	1038	398	1125	398
12	407	387	329	387	636	387	646	388	836	387	1324	387
13	388	406	398	406	393	406	897	407	944	407	929	407
14	449	428	452	428	805	428	818	428	845	429	877	429
15	286	263	296	264	349	263	616	264	640	264	734	264
16	388	358	397	358	730	358	872	359	836	359	909	359

i. s.: inoculated side of the udder, c. s.: uninoculated side of the udder.

Somatic cell counts expressed as N × 10^3^ cells mL^−1^.

**Table 4:** Detailed data of mastitis presence in ewes after intramammary inoculation (performed on D0, after the respective sampling).

Ewe no.	D0		D0 + 12 h		D1		D2		D3		D4	
	i. s.	c. s.	i. s.	c. s.	i. s.	c. s.	i. s.	c. s.	i. s.	c. s.	i. s.	c. s.
(a) Experiment 1 (deposition of *M. haemolytica* into the teat duct)												
1	–	–	–	–	+	–	+	–	+	–	+	–
2	–	–	–	–	+	–	–	–	–	–	NA	NA
3	–	–	+	–	+	–	+	–	+	–	–	–
4	–	–	–	–	+	–	+	–	–	–	NA	NA
5	–	–	–	–	+	–	–	–	–	–	NA	NA

Ewe no.	D0		D0 + 3 h		D0 + 6 h		D0 + 9 h		D0 + 12 h		D1	
	i. s.	c. s.	i. s.	c. s.	i. s.	c. s.	i. s.	c. s.	i. s.	c. s.	i. s.	c. s.
(b) Experiment 2 (inoculation of *M. haemolytica* [ewes 11–13] or *S. chromogenes* [ewes 14–16] into the mammary gland cistern)												
11	–	–	–	–	+	–	+	–	+	–	+	–
12	–	–	–	–	+	–	+	–	+	–	+	–
13	–	–	–	–	–	–	+	–	+	–	+	–
14	–	–	–	–	+	–	+	–	+	–	+	–
15	–	–	–	–	–	–	+	–	+	–	+	–
16	–	–	–	–	+	–	+	–	+	–	+	–

i. s.: inoculated side of the udder, c. s.: uninoculated side of the udder.

+: presence of mastitis, -: no mastitis.

Mastitis definition: mastitis was defined in ewes with (i) clinically evident abnormalities in mammary gland or mammary secretion or (ii) with no clinical abnormalities, but in which a bacteriologically positive milk sample with concurrently increased cell content (CMT score ≥ ‘l’ or cell counts ≥ 0.5 × 106 cells mL-1) plus increased neutrophil and lymphocyte proportion (≥65% of all leucocytes) in Giemsa-stained milk films was detected.

**Table 5:** Detailed data of densities of spots corresponding to cathelicidin-1 on 2-DE gels prepared from milk whey samples from ewes after intramammary inoculation (performed on D0, after the respective sampling).

Ewe no.	D0		D0 + 12 h		D1		D2		D3		D4	
	i. s.	c. s.	i. s.	c. s.	i. s.	c. s.	i. s.	c. s.	i. s.	c. s.	i. s.	c. s.
(a) Experiment 1 (deposition of *M. haemolytica* into the teat duct)												
1	0	0	282	0	88	27	0	0	7	0	0	0
	0	0	60	0	0	0	0	0	0	0	0	0
2	0	0	878	0	142	4	123	0	117	13	NA	NA
	0	0	152.6	0	0	0	4	0	17	16		
	0	0	55	0	11	0	0	0	0	0		
3	0	0	8638	0	8463	309	1470	74	750	0	3005	0
	0	0	2627	0	1734	0	0	0	0	0	141	0
	0	0	175	0	119	0	0	0	0	0	0	0
	0	0	2521	0	654	0	0	0	0	0	0	0
	0	0	139	0	2	0	0	0	0	0	0	0
4	0	0	164	0	17	0	0	0	2	0	NA	NA
5	0	0	820	0	810	0	562	0	1259	0	NA	NA
	0	0	278	0	0	0	0	0	493	0		

Ewe no.	D0		D0 + 3 h		D0 + 6 h		D0 + 9 h		D0 + 12 h		D1	
	i. s.	c. s.	i. s.	c. s.	i. s.	c. s.	i. s.	c. s.	i. s.	c. s.	i. s.	c. s.
(b) Experiment 2 (inoculation of *M. haemolytica* [ewes 11–13] or *S. chromogenes* [ewes 14–16] into the mammary gland cistern)												
11	0	0	107	0	1437	0	230	0	444	0	121	0
	0	0	5	0	230	0	39	0	28	0	0	0
12	0	0	0	0	212	0	1658	0	3496	0	5135	0
	0	0	0	0	0	0	0	0	1482	0	170	0
	0	0	0	0	0	0	152	0	780	0	1468	0
13	0	0	0	0	0	0	2095	0	1326	0	0	0
	0	0	298	0	4817	0	8313	0	5244	0	623	0
	0	0	0	0	601	0	1011	0	826	0	0	0
	0	0	0	0	990	0	752	0	1673	0	0	0
14	0	0	95	0	498	0	1733	0	3951	0	781	0
	0	0	0	0	276	0	565	0	732	0	165	0
15	0	0	0	0	213	0	636	0	835	0	326	0
	0	0	76	0	351	0	1051	0	640	0	0	0
16	0	0	61	0	810	0	4075	0	1381	0	426	0

i. s.: inoculated side of the udder, c. s.: uninoculated side of the udder.

**Table 6:** Cumulative data (mean ± standard error of the mean) of densities of spots corresponding to cathelicidin-1 on 2-DE gels prepared from milk whey samples from ewes after intramammary inoculation (performed on D0, after the respective sampling).

Udder side	Total spots (n)	Before challenge	After challenge				
		D0	D0 + 12 h	D1	D2	D3	D4
(a) Experiment 1 (deposition of *M. haemolytica* into the teat duct)							
Inoculated	2.6 ± 0.7^a^	0.0 ± 0.0^x^	3357.9 ± 2687.6^a,k,l,x^	2408.3 ± 2142.0^a,k,m,x^	431.7 ± 278.9^l,m^	529.0 ± 334.8^a,x^	1573.1 ± 1577.8
Un-inoculated	0.8 ± 0.4^a^	0.0 ± 0.0	0.0 ± 0.0^a^	68.2 ± 60.3^a^	14.8 ± 14.7	5.7 ± 5.7^a^	0.0 ± 0.0

Udder side	Total spots (n)	Before challenge	After challenge				
		D0	D0 + 3 h	D0 + 6 h	D0 + 9 h	D0 + 12 h	D1
(b) Experiment 2 (inoculation of *M. haemolytica* or *S. chromogenes* into the mammary gland cistern)							
Inoculated	2.3 ± 0.4	0.0 ± 0.0^x^	89.1 ± 44.6	1499.8 ± 987.0^a^	3679.8 ± 1778.0^a,x^	3732.0 ± 1387.9^a,x^	1515.7 ± 1059.1^a,x^
Un-inoculated	0.0 ± 0.0	0.0 ± 0.0	0.0 ± 0.0	0.0 ± 0.0^a^	0.0 ± 0.0^a^	0.0 ± 0.0^a^	0.0 ± 0.0^a^

Within each experiment: a = *P* ≤ 0.05 between inoculated and uninoculated glands, k-m = *P* ≤ 0.05 between inoculated glands and x = *P* ≤ 0.05 for inoculated glands compared to D0.

**Fig. 5:**
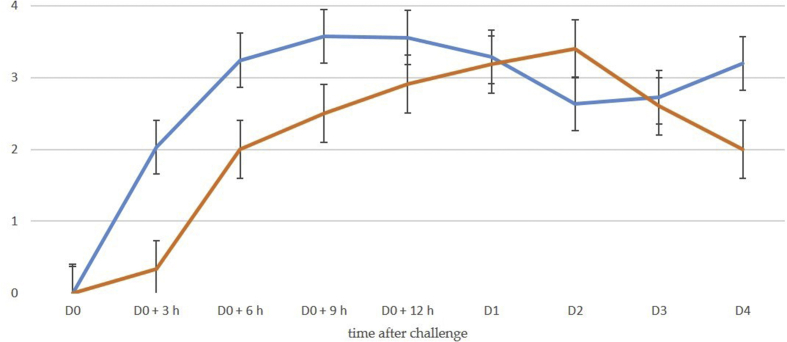
Log_10_ of mean spot densities of cathelicidin-1 in 2-DE gels (blue line) and mean CMT scores (brown line) in sequential milk samples from inoculated side of the udder, subsequently to intramammary infection (experiments 1 and 2). Experiment 1: deposition of *M. haemolytica* into the teat duct, experiment 2: inoculation of *M. haemolytica* or *S. chromogenes* into the mammary gland cistern.

**Fig. 6:**
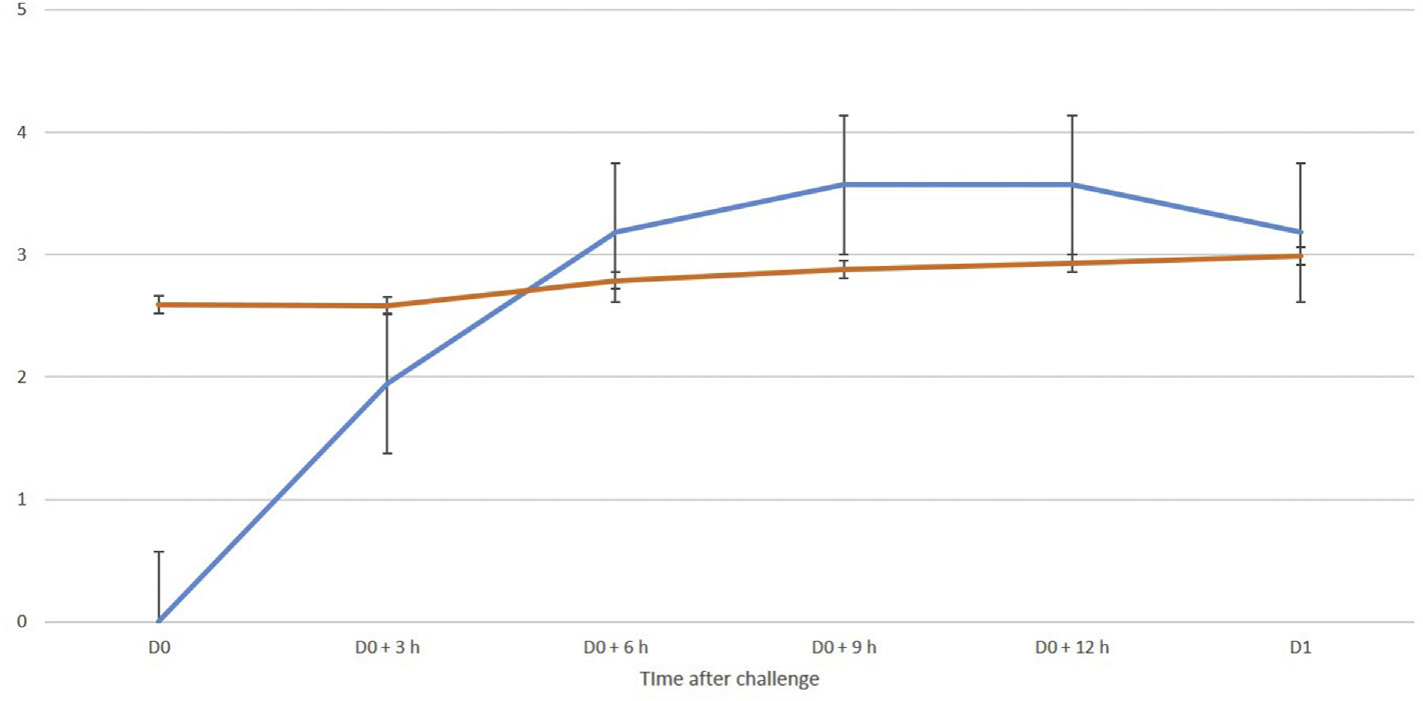
Log_10_ of mean spot densities of cathelicidin-1 in 2-DE gels (blue line) and log_10_ of mean somatic cell counts (brown line) in sequential milk samples from inoculated side of the udder, subsequently to intramammary infection with *M. haemolytica* (experiment 2).

The data have provided evidence of a significant association between cell content and detection of cathelicidin-1 in respective milk sample (Fig. 5, Fig. 6), as well as between presence of mastitis in a mammary gland and detection of cathelicidin-1 in the respective milk sample (Table 7). The association was stronger in samples collected during the first 24 h post-inoculation than in samples collected thereafter (Table 8). There was a slight increase in cathelicidin-1 levels, when a higher challenge dose of *M. haemolytica* was used (Experiment I).

**Table 7:** 2 × 2 contingency table indicating number of milk samples from mammary glands with mastitis (‘positive’ [+] or ‘negative’ [−]) in relation to detection of cathelicidin-1 therein (‘positive’ [+] or ‘negative’ [−]) (experiments 1 and 2).

		Cases of mastitis	
		+ (n = 34)	+ (n = 34)
Detection of cathelicidin-1	+ (n = 53)	31	20
	- (n = 73)	3	72

Experiment 1: deposition of *M. haemolytica* into the teat duct, experiment 2: inoculation of *M. haemolytica* or *S. chromogenes* into the mammary gland cistern.

**Table 8:** 2 × 2 contingency tables indicating number of milk samples from mammary glands with mastitis (‘positive’ [+] or ‘negative’ [−]) in relation to detection of cathelicidin-1 therein (‘positive’ [+] or ‘negative’ [−]).

		Cases of mastitis	
		+ (n = 12)	+ (n = 42)
(a) Experiment 1 (deposition of *M. haemolytica* into the teat duct)			
Detection of cathelicidin-1	+ (n = 22)	9	13
	- (n = 32)	3	29

		Cases of mastitis	
		+ (n = 22)	+ (n = 50)
(b) Experiment 2 (inoculation of *M. haemolytica* or *S. chromogenes* into the mammary gland cistern)			
Detection of cathelicidin-1	+ (n = 29)	22	7
	- (n = 43)	0	43

Data indicated a correlation between CMT scores and cathelicidin-1 spot densities in milk samples: the correlation coefficient for both experiments was *r* = 0.398 (*P* < 0.001); the respective values for experiments 1 and 2 were *r* = 0.272 (*P* = 0.023) and *r* = 0.540 (*P* < 0.001). In experiment 2, the correlation coefficients when data from ewes inoculated with *M. haemolytica* or *S. chromogenes* were taken separately were *r* = 0.604 and 0.704 (*P* < 0.001), respectively. There was also evidence of correlation between somatic cell counts and cathelicidin-1 spot densities in milk samples in experiment 2. The correlation coefficient was *r* = 0.565 (*P* < 0.001).

## 2 Experimental design, materials and methods

In experiment I, *M. haemolytica* (1000–1250 c.f.u.) was deposited into the teat duct of ewes (n = 5) on Day 0 (D0). In experiment II, *M. haemolytica* (50–80 c.f.u.) or *S. chromogenes* (1 × 10^6^–2 × 10^6^ c.f.u) was inoculated into in the gland cistern of ewes (n = 3 for each pathogen) also on D0. In all cases, mastitis was induced, as confirmed by microbiological and cytological examination of milk samples, which were collected on D0 + 12 h, D1, D2, D3 and D4 (experiment 1) or on D0 + 3 h, D0 + 6 h, D0 + 9 h, D0 + 12 h and D1 (experiment 2). The uninoculated mammary gland (contralateral) was used as uninfected control. Increased cell content and recovery of the challenge pathogens were simultaneously recorded. Milk whey prepared from the samples was processed for proteomics examination.

Proteomics analysis for detection of cathelicidin-1 was performed as detailed by Katsafadou et al. (2019). Two-dimensional gel electrophoresis was used initially.

In experiment 1, image analysis was performed as detailed by Katsafadou et al. (2019) and included all the surface of each gel; spots corresponding to cathelicidin-1 were identified. In experiment 2, image analysis was limited in the region of each gel, where cathelicidin-1 had been located during experiment 1. Spot optical densities obtained from PD Quest v.8.0 for each spot of interest on each gel on D0 or sequentially after challenge, were recorded. In case of multiple spots indicative of the same protein, densities of all spots were taken into account. The spot volume was used as the analysis parametre to quantify protein expression.

Protein identification was performed by peptide mass fingerprinting. Peptide mixtures were analysed in a MALDI-TOF MS (Matrix-Assisted Laser Desorption/Ionization Time-Of-Flight Mass Spectrometer) (Ultraflex, Bruker Daltonics). Matching of peptides and protein searches were carried out in the MASCOT Server 2 (Matrix Science, Boston, USA). Full details of the procedure have been presented by Katsafadou et al. [2].
